# The Speed of Smell: Odor-Object Segregation within Milliseconds

**DOI:** 10.1371/journal.pone.0036096

**Published:** 2012-04-27

**Authors:** Paul Szyszka, Jacob S. Stierle, Stephanie Biergans, C. Giovanni Galizia

**Affiliations:** Department of Biology, University of Konstanz, Konstanz, Germany; Center for Genomic Regulation, Spain

## Abstract

Segregating objects from background, and determining which of many concurrent stimuli belong to the same object, remains one of the most challenging unsolved problems both in neuroscience and in technical applications. While this phenomenon has been investigated in depth in vision and audition it has hardly been investigated in olfaction. We found that for honeybees a 6-ms temporal difference in stimulus coherence is sufficient for odor-object segregation, showing that the temporal resolution of the olfactory system is much faster than previously thought.

## Introduction

Most natural odors consist of many components, though they are perceived as unitary odor-objects [1]. Because airborne odorants intermingle and fluctuate at fast timescales [2], [3], the olfactory system needs to segregate concurrent odors from independent sources in order to recognize them as different odor-objects [4], [5]. This problem is analogous to figure-ground segregation in vision [6] and concurrent sound segregation in audition [7]. Both, the visual and auditory system analyze temporal coherence between stimuli for object segregation [7], [8]. It is not known whether odor-object segregation is also based on temporal stimulus coherence. Studies on mixture processing in honeybees and other species demonstrated that mixtures have a perceptive quality that is different from their components [9]–[14], thus making it difficult to recognize odor-objects from mixtures. These studies only considered static step like stimuli. Rapid odorant fluctuations, however, contain information that can be used for odor-source tracking [15]–[18]. Accordingly, information contained in the fast temporal structure of odorant stimuli might be used to segregate an odor-object from a mixture [4], [5], [19].

To address this idea, we asked whether honeybees can use short temporal differences between two components of a binary odorant mixture to extract information about its components. We first trained honeybees to respond to an odorant *A* by pairing *A* with a sugar reward [20]. Then, we tested memory retrieval with a mixture of *A* and a novel odorant *B*. We found that a 6 ms asynchrony in the onset of *A* and *B* is sufficient to enhance the salience of the component odor information, and that it is not necessary that the component in question was presented alone at any time during the stimulus.

## Results

**Figure 1:**
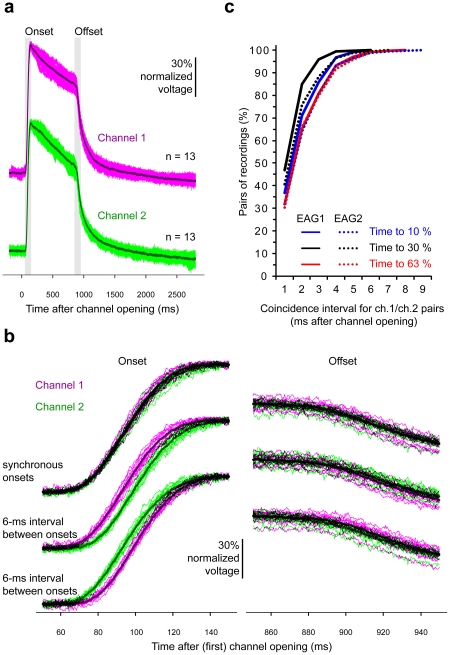
Temporal characteristics of the odorant stimuli. (**a**) Electroantennogram (EAG) response to odorant stimuli delivered by channel 1 (magenta, shifted up for clarity) and channel 2 (green) of the olfactometer. 13 single measurements and superimposed mean (dark trace). Stimulus duration was 800 ms. Channel 1 and 2 were measured sequentially. (**b**) Blow-up of the stimulus onset and offset (shaded period in (a)), shifted vertically for clarity. Top: Channel 1 and 2 opened and closed simultaneously (data from (a)). Middle: Channel 2 opened and closed 6 ms after channel 1. Bottom: Channel 1 opened and closed 6 ms after channel 2. N = 13 measurements each. To detect possible mechanical effects of opening two channels in the incoherent mixture, a blank channel was opened 6 ms before or after the opening of the tracer channel (middle: blank opened 6 ms after channel 1 or 6 ms before channel 2, bottom: vice versa). All traces were normalized to the amplitude maximum. (**c**) Percentage of EAG recordings for pairs of channel 1 and 2 that reached either 10, 30 or 63% of the amplitude maximum within a given coincidence interval. EAG1 (26 recordings per channel, 676 pairs, same data as in (a) and (b)) and EAG2 (28 recordings per channel, 784 pairs) show data from two independent EAG recordings.

Studying the effect of millisecond time-differences in stimulus coherence on the perception of odorant mixtures requires temporally precise odorant stimuli. In our experiments, we mixed two odorants with an onset or offset delay of 6 ms. We therefore tested the temporal precision of odorant delivery in this time range using electroantennogram (EAG) recordings. Odorant stimuli evoked EAG responses with fast and reproducible response dynamics (**Figure 1a**). The rise time (10 to 90%) was less than 50 ms, and the difference in reaching 30% of amplitude maxima between two odor channels was 0.4±2 ms (mean ± standard deviation) (**Table S1**). The 6-ms interval between the opening of channel 1 and 2 used for our mixture experiments was clearly visible in the onset of the EAG responses (**Figure 1b**). The offset, however, was less precise and the 6-ms interval could not reliably be reproduced. When opening channel 1 and 2 simultaneously more than 40% of the EAG signals coincided within 1 ms and more than 75% coincided within 2 ms in reaching 30% of the maximum (**Figure 1c**).

**Figure 2:**
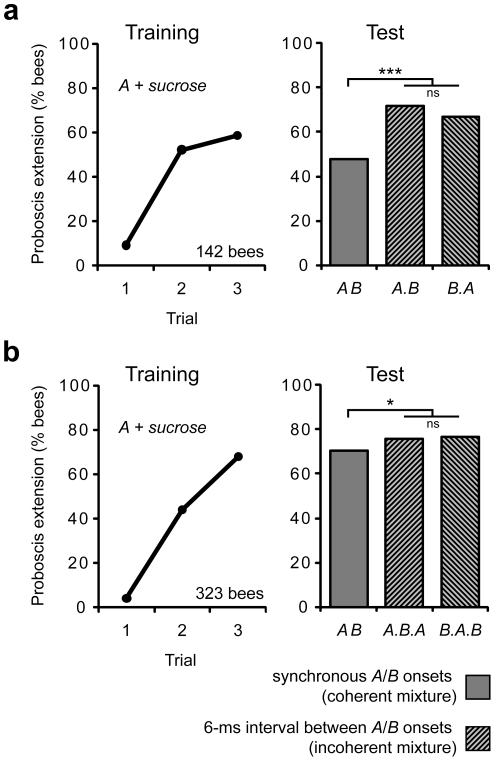
A 6-ms temporal difference in stimulus coherence is sufficient for odor-object segregation. (**a**) Each bee received 3 rewarded training trials with *A*, and the percentage of bees showing odor-evoked proboscis extension is shown. Odorant stimulus duration was 800 ms. During the memory test, odorants *A* and *B* were presented simultaneously (coherent mixture, *AB*) or with a 6-ms interval between their onsets (incoherent mixture). One incoherent mixture started with *A* (*A.B*), the other with *B* (*B.A*). Test stimulus sequence was randomized. The proboscis extension rate for the incoherent mixtures was higher than for the coherent mixture (one-way RM ANOVA; F(2, 425) = 17.1, p<0.001, Holm-Sidak posthoc test; N = 142). (**b**) Same experimental protocol as in (a) but odorant *A* was presented against the background of odorant *B* (*B.A.B*) and odorant *B* against odorant *A* (*A.B.A*). Background-odorant lasted 806 ms, starting 6 ms before and stopping 6 ms after the 794-ms long foreground-odorant. The proboscis extension rate for the incoherent mixtures was higher than for the coherent mixture (F(2, 968) = 4.7, p<0.01; N = 323). Experiments in (a) and (b) were done at different times of the year, and the response difference during training and testing to *AB* might reflect seasonal differences in learning and memory performance. ***, p<0.001; *, p<0.02.

Bees were trained to associate an odorant *A* with a sugar reward, learning to extend their mouthparts (proboscis) in response to the odorant and in anticipation of the reward (3 trial classical conditioning, **Figure 2**). Thirty minutes after training, odorant *A* was presented in temporally coherent (synchronous odorant onset and offset) or incoherent (asynchronous odorant onset and offset, 6 ms delay) mixtures with a new odorant *B*. How much a bee “recognized” *A* in the mixture was assessed by its proboscis extension response. We first tested whether a 6-ms interval between the on- and offsets of *A* and *B* would facilitate their segregation from the mixture (**Figure 2a**). Bees' response rates to the incoherent mixtures *A.B* (odorant *A* first) and *B.A* (odorant *B* first) were significantly higher than to the coherent mixture *AB*. Interestingly, there was no statistically significant difference between *A.B* and *B.A*. This data suggests that bees either use temporal incoherence or the 6-ms presence of a pure odorant, or both to segregate a component odorant from a mixture. To distinguish these alternative explanations, we modified the test and presented either *A* against the background of *B* (*B.A.B*; *B* onset 6 ms before *A* onset, *A* offset 6 ms before *B* offset) or *B* against the background of *A* (*A.B.A*) (**Figure 2b**). Most bees which learned during the training did not discriminate between the coherent mixture *AB* and the incoherent mixtures *A.B.A* and *B.A.B*, and 79% responded equally to the three mixtures. However, the response rates for the incoherent mixtures were higher than for the coherent mixture. Again, we found no difference between *A.B.A* (a situation where, for 6 ms, *A* could be smelled alone), and *B.A.B* (a situation where *A* is never presented alone).These results indicate that bees use temporal incoherence rather than the 6-ms presence of a pure odorant for odor-object segregation.

## Discussion

One of the most intriguing capacities of our brain is the so-called cocktail party effect: the possibility to extract the voice of our conversation partner amidst a cacophony of different voices and sounds. This is particularly impressive given the strong overlap in the frequency range, and hence the receptor neuron activation, of the different sound sources that the brain is able to segregate. It is believed that this capacity of the brain is based on an analysis of the fine-scale temporal structure and coherence of the different sources [7]. Similar effects have been shown for the visual modality, in particular for object segregation in dynamical visual fields [8]. In the acoustic system of humans, delays of 30 ms are sufficient to hear that two different sources are causing a sound [21], while the human visual system requires delays of 6 ms for figure-ground segregation [22]. Even though physiological responses to odor-mixtures with asynchronous onset has been studied to some extent [23], and the dynamical response properties of olfactory receptor neurons are known for some species [24]–[27], only one behavioral study about dynamical odor-object segregation is known to us [19].

After conditioning to respond to an odorant *A*, honeybees were more likely to respond to a mixture of *A* and a novel odorant *B* if the onsets of *A* and *B* were shifted by 6 ms. From this result we conclude that the short time difference between the onsets of two overlapping odorant stimuli facilitates their segregation. An alternative conclusion would be that the 6-ms time difference between odorant stimuli increases the mixtures' saliency due to mechanical interference between the channels of the olfactometer. We therefore took great care in designing an olfactometer that produces odorant pulses free of mechanical interferences [28]. The opening of an empty channel 6 ms before or after an odor channel did not produce any visible disturbance in EAG recordings (**Figure 1b**).

We conclude that honeybees can detect temporal incoherence between odorant stimuli in the millisecond range and use this information to extract odorants' identity. This seems a remarkable performance considering that the sense of smell is regarded to be a relatively slow sense as compared to the auditory or visual senses. Odor discrimination tasks in different species showed that 200 to 600 milliseconds are required for odor recognition [29]–[31]. Thus, the insect olfactory system reveals a hitherto unknown fast-processing property. Our findings open new perspectives for the study of odor-object perception, and suggest mechanisms that allow us to recognize a whiff of perfume in a mall full of other odorants.

It will be interesting to examine the physiological mechanisms underlying odor-object segregation. In *Drosophila* olfactory receptor neurons can encode the dynamics of odorants that fluctuate as fast as 100 Hz [26], [32], [33], and in locust neural representations of mixtures partly match those evoked by the individual components if their onsets differ by 100 ms [23]. It remains to be shown whether this also holds true for the bee and for onset-differences of just a few milliseconds. Olfactory coding follows similar rules across animal species from mammals to insects [34]. Therefore, these mechanisms might be generalizable to mammalian olfaction, another hypothesis that remains to be tested. Moreover, they could be used to develop control algorithms for autonomous odor-source tracking robots.

## Materials and Methods

We used 1-hexanol and 1-nonanol (diluted 1∶100 in mineral oil; all from Sigma-Aldrich) as odorant stimuli. 1-hexanol and 1-nonanol were equally often used as odorant *A* and odorant *B*. As a reward during training we presented a 3-s long sucrose stimulus (1 M in water) which started 1.2 s after odorant offset. The intertrial interval was 10 minutes. Thirty minutes after the end of training odorant *A* was presented in temporal coherent and incoherent mixtures with a new odorant *B*. The sequence of the mixture stimulation was balanced across bees to exclude sequence-effects, and the experimenter was blind for the stimulus identity.

The olfactometer consisted of three channels. Through each channel air (300 ml/min) was injected into a carrier air stream (2100 ml/min). During the conditioning experiments, channel 1 was used for 1-nonanol and channel 2 for 1-hexanol. The exit diameter of the olfactometer was 6.8 mm, resulting in airspeed of 138 cm/s. Bees were placed 2 cm in front of the olfactometer. A more detailed description of the olfactometer and conditioning procedure is given in [28].

The temporal characteristics of the odor stimuli were measured with electroantennogram (EAG) recordings 2 cm in front of the olfactometer (7 cm away from where the channels are injected into the carrier airstream). Two EAG recordings were done, each with a single bee antenna (EAG1, EAG2). 10 µl of pure 2-heptanone was used as tracer odorant. The 4 different stimuli (channel 1 and channel 2; 0 and 6 ms delays) were presented in an alternating sequence and the interstimulus interval was 30 s. For EAG recordings a single antenna was cut in the middle of the scapus and was mounted with conductive gel (GEL+, Ritex) between the two poles of a stainless steel electrode (Kombi PROBE, Syntech). The signal was band-pass filtered for the 0.1 Hz to 3 kHz range (AM 502, Tektronix) and digitized at a sampling rate of 2500 Hz (Digidata 1200, Axon Instruments). EAG signals were normalized to the amplitude maximum to correct for changes in response strength and the baseline was shifted to zero to correct for baseline drifts. Data was analyzed with R (www.r-project.org). Similar measurements were done with a photoionization device [35] to exclude biological influences, with comparable results (data not shown).

## Supporting Information

Table S1 **Temporal characteristics of EAG responses. Time intervals between channel openings and reaching 10, 30 or 63% of amplitude maxima, and rise time, measured as time required for the EAG to rise from 10 to 90% (means and standard deviation, all data in ms).** EAG1 and EAG2 are two EAG recordings (same as in Fig. 1). The differences are calculated for all possible pairs of channel 1and 2 (EAG1: 26 recordings per channel, 676 pairs; EAG2: 28 recordings per channel, 784 pairs).
(TIF)
