# Supplementary figures and images for: The Speed of Smell: Odor-Object Segregation within Milliseconds
Source: PLoS One. 2012 Apr 27;7(4):e36096. doi: 10.1371/journal.pone.0036096 (PMC3338635; doi:10.1371/journal.pone.0036096)

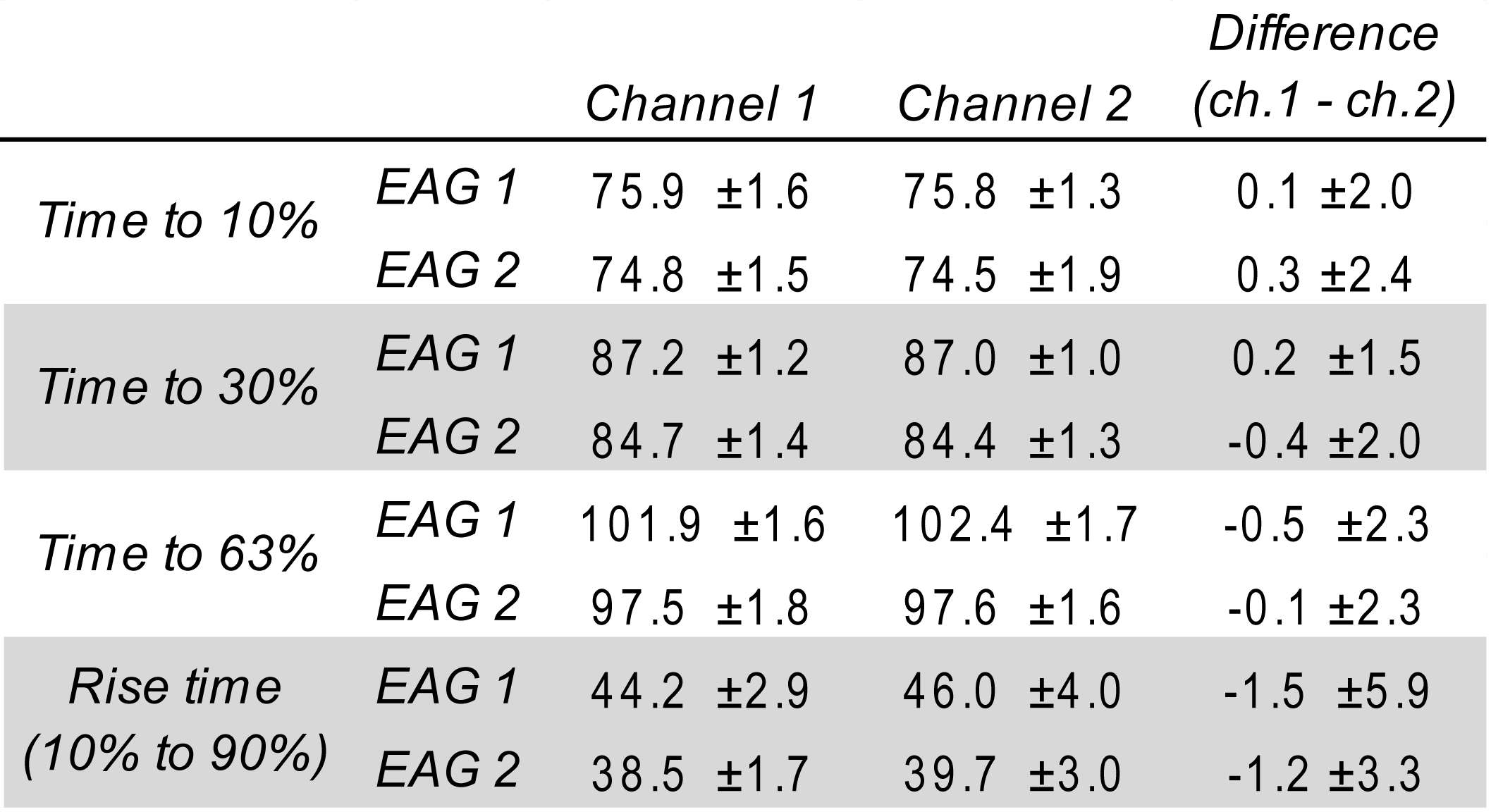

Supplement: Table S1 — Temporal characteristics of EAG responses. Time intervals between channel openings and reaching 10, 30 or 63% of amplitude maxima, and rise time, measured as time required for the EAG to rise from 10 to 90% (means and standard deviation, all data in ms). EAG1 and EAG2 are two EAG recordings (same as in Fig. 1). The differences are calculated for all possible pairs of channel 1and 2 (EAG1: 26 recordings per channel, 676 pairs; EAG2: 28 recordings per channel, 784 pairs). (TIF) [file pone.0036096.s001.tif]
